# The PD-1 and CD28 molecules on T cells in peripheral blood are associated with the prognosis of patients with advanced breast cancer receiving paclitaxel chemotherapy

**DOI:** 10.1371/journal.pone.0344366

**Published:** 2026-04-06

**Authors:** Ji Yang, Shuxian Qu, Xinhui Qi, Yongming Liu, Jianing Qiu, Ying Yu, Yino Wang, Zhendong Zheng, Huiying Yu

**Affiliations:** 1 Basic Medicine Laboratory, General Hospital of Northern Theater Command, Shenyang, China; 2 Department of Clinical Oncology, General Hospital of Northern Theater Command, Shenyang, China; 3 Department of Breast Surgery, the First Affiliated Hospital of China Medical University, China; 4 Department of Breast Surgery, Anshan Tumor Hospital, China; University of Missouri, UNITED STATES OF AMERICA

## Abstract

**Background:**

T cells are the primary cell subset involved in antitumor immunity. Their functions depend largely on the repertoire of surface receptors. This study investigates the association between the expression of CD28 and PD-1 molecules on peripheral blood T cells and prognosis in advanced breast cancer (BC) patients undergoing paclitaxel chemotherapy, along with their roles in anti-tumor immunity.

**Methods:**

Peripheral blood from 61 patients with advanced BC was analyzed by flow cytometry for immunophenotype before treatment. We investigated the effects of PD-1 and CD28 on anti-tumor responses of lymphocytes in two breast cancer cell lines in vitro.

**Results:**

Patients with high CD28 and low PD-1 on T cells were more sensitive to chemotherapy. PD-1 blockade enhanced the cytotoxicity of peripheral blood mononuclear cells (PBMCs) against tumor cells with high expression of PD-L1, an effect dependent on CD28 expression on T cells. CD28 expression is associated with immune cell infiltration in BC tumor microenvironment, particularly CD8^+^ T effector memory (Tem) cells. The proportion of CD8^+^ Tem cells increased after treatment with anti-PD-1 antibodies in vitro. Clinical results show that most patients who do not respond to chemotherapy have high expression of CD3^+^PD-1^+^ and low expression of CD8^+^CD28^+^. Combining the two markers distinguished patients with progressive disease more accurately. Additionally, patients with low CD3^+^PD-1^+^ expression and high CD8^+^CD28^+^ expression had longer progression-free survival (PFS).

**Conclusion:**

The expression of CD28 and PD-1 on T cells can serve as potential biomarkers for assessing tumor progression, chemotherapy response and prognosis in advanced BC. BC patients with low PD-1 expression or PD-1 blockade exhibit increased induction of memory T cells during the anti-tumor immune response.

## Introduction

Breast cancer (BC) is the most common malignant disease among women worldwide. Approximately 70–80% of early-stage patients achieve long-term survival. The primary goals of advanced (metastatic) disease treatment are to prolong survival, control symptoms, reduce treatment-related toxicity and maintain or improve quality of life [1]. Paclitaxel (PTX) is frequently used as a first-line chemotherapy agent for BC, especially in advanced metastatic cancer. Tumor progression is modulated by the host immune system. Many tumor patients suffer from immune dysfunction. Successful antitumor immunity depends on the ability of effector immune cells to recognize and attack tumor cells and enhance other immune cells [2].

T lymphocytes are the major antitumor immune cells. Detecting T cell subsets is an important method for assessing cellular immunity function. CD28 is a co-stimulator of T cell activation and survival. As T cells undergo antigen-mediated activation and differentiation, they gradually lose CD28 expression. The increase in age-related CD28^-^ T cells in peripheral blood indicates that immune function is impaired in elderly individuals [3]. Among CD28^-^ T cell subsets, CD8^+^CD28^-^ T cells show the greatest correlation with the age growth [4]. Recently, these cells have attracted interest due to their role in immune regulation. Accumulating evidence shows that CD8^+^CD28^-^ T cells are a distinct subset of regulatory T cells with direct and broad effects on other T cells, including inhibition of their activation and proliferation, reduction the secretion of proinflammatory cytokines by activated T cells, and induction activated T cells apoptosis in vitro. The number of CD8^+^CD28^-^ suppressor T cells increases in cancers, viral infections, autoimmune diseases and nearly all chronic inflammatory conditions. Their expansion is associated with disease progression [5,6]. CD28/CD80 is the second signal for T cells activation. PD-1/PD-L1 is an inhibitory signal that acts as an immune “brake”. Currently, anti-PD-1/PD-L1 immune checkpoint blockade (ICB) therapy is widely used in clinical oncology and offers clinical benefits. The therapy primarily restores exhausted CD8^+^ T cells by blocking PD-1/PD-L1 signaling. PD-1/PD-L1 ICB has become an important strategy for treating malignant tumors [7,8]. However, unlike with other malignancies such as lung cancer, immunotherapy has progressed more slowly with BC. Current research primarily focuses on triple-negative breast cancer (TNBC), for with clinical benefits remain limited [9–12].

In this study, we evaluated the functional status of T cells by observing the expression of CD28 and PD-1 on peripheral blood T cells from patients with advanced BC undergoing paclitaxel chemotherapy. We also analyzed the correlation of these markers with tumor occurrence and development, treatment response and patient prognosis.

## Methods

### Patient selection and sample collection

This study was approved by the Ethics Committee of the General Hospital of Northern Theatre Command. Patients provided their written informed consent. We enrolled BC patients admitted to our hospital between November 25, 2019 and March 30, 2021. Inclusion criteria were as follows: 1) Pathologically confirmed advanced BC (stage III or IV) prior to treatment; 2) No anticancer therapy had been received within 6 months prior to enrollment; 3) Receiving chemotherapy regimens including paclitaxel; 4) Age > 18 years. Exclusion criteria were as follows: 1) Patients with a history of other tumors or other malignancies/infections/autoimmune diseases/blood diseases, etc.; 2) receiving immunosuppressive therapy; 3) With drug-dependence/uncontrolled mental illness/cognitive dysfunction; 4) During pregnancy/lactation. Clinical approximation of BC subtypes were defined using the St. Gallen Criteria [13].

### Peripheral blood mononuclear cells isolation

Peripheral blood mononuclear cells (PBMCs) were isolated by Ficoll density gradient centrifugation. For each patient, 10 mL of peripheral blood was collected in a heparinized tube. The samples were processed and diluted 1:1 with an equal volume of phosphate buffered saline (PBS). The diluted blood was carefully layered on the top of the Ficoll-Hypaque solution in 50 mL centrifuge tubes. After centrifugation at 800 × g for 20 min without brake, the PBMCs were collected from the interface layer, transferred to a new centrifuge tube, and washed with PBS.

### Cell lines

MCF-7 and MDA-MB-231 breast cancer cell lines (both of which are metastatic adenocarcinoma cell lines obtained from ATCC, Manassas, VA, USA) were used. MCF-7 cells were cultured in DMEM (BasalMedia Co., China) supplemented with 10 μg/mL of insulin. MDA-MB-231 cells were cultured in Leibovitz’s L-15 medium (BasalMedia Co., China). All media contained with 10% (v/v) heat-inactivated fetal bovine serum (FBS) (VivaCell, China). Cells were cultured at 37°C in 5% CO_2_. The study only used cells from passages 4 and 5.

### Co-cultures of MCF-7 and MDA-MB-231 cells with PBMCs

MCF-7 and MDA-MB-231 cell lines were co-cultured with PBMCs from BC patients. MCF-7 and MDA-MB-231 cells were seeded at a density of 5 × 10^4^ cells/well in a 24-well plate and allowed to attach for 12 h. PBMCs were stimulated with 100 ng/mL of human CD3 antibody (clone OKT3) (BioLegend, USA) and 1000 IU/mL interferon (IFN)-γ 24h. Then, they were placed on MDA-MB-231 or MCF-7 cells (5 × 10^5^ cells per well) and incubated for the next 24 h at 37°C in a 5% CO_2_ atmosphere. PBMCs were pre-treated with 3 µg/mL anti-PD-1 polyclonal antibody (Camrelizumab, Hengrui Pharmaceuticals Co., China) for 30 min prior to co-culturing in the experimental groups.

MCF-7 and MDA-MB-231 were seeded a density of 5 × 10⁴ cells/well in 24-well cell culture plates. The target-to-effector cell ratio was 10:1. The cells were co-cultured for 24 h and recollected for further studies.

### Immunophenotyping of lymphocyte populations

Blood samples from the BC patients were collected in ethylenediaminetetraacetic acid (EDTA) anticoagulant tubes prior to treatment. Peripheral lymphocytes or PBMCs were stained with surface markers using the following specific anti-human monoclonal antibodies for 30 min in the dark, at room temperature: CD4 – fluorescein isothiocyanate (FITC) (clone: SK3), CD8 – phycoerythrin (PE) (clone: SK1), CD3 - peridinin-phlorophyll-protein complex (PerCP) (clone: SK7), CD56 – allophycocyanin (APC) (clone: NCAM16.2), CD28 – PE-cyanine (Cy) 7 (clone: CD28.2), CD62L - APC (clone: DREG-56), CD45RA - PE-Cy7 (clone: L48), CD38 – APC (clone: HB-7), HLA-DR – APC-Cy7 (clone: L243) (the above antibodies were purchased BD Biosciences, USA)，PD-1 – APC (clone: J105) (eBiosciences, USA) and granzyme B (GzmB) – PE-Cy7 (clone: QA16A02) (BioLegend, USA). Add 100 μL of anticoagulated blood or least 5 × 10^5^ PBMCs to each tube and mix thoroughly. Red blood cell lysis was performed using 1 mL of 1 × BD FACS Lysing Solution (BD Biosciences, USA). Intracellular proteins were permeabilized with 1 × Fixation and Permeabilization Solution and then stained with anti-GzmB. Flow cytometry (FACS Canto Ⅱ, BD Biosciences, USA) was used to analyze the residual lymphocytes or PBMCs. Gating strategies for the flow cytometric analysis were shown in S1 Fig.

### Evaluation of apoptosis by flow cytometry

After co-culturing tumor cells with PBMCs for 24 hours, the cells were incubated with trypsin until 80% of them were detached. Annexin V-FITC/propidium iodide (PI) double labeling was used to evaluate the apoptosis rate of MCF-7 and MDA-MB-231 cells according to the manufacturer’s instructions (Biosharp, China). Flow cytometry was used to analyze those cells. Compare the cytotoxic effects of PBMCs with and without anti-PD-1 through the cell apoptosis rate. The growth rate of the killing ability was calculated as follows: [(100% - living cells% in anti-PD-1) – (100% - living cells% without anti-PD-1)]/ (100% - living cells% without anti-PD-1).

### Prognosis assessment

The efficacy assessment was based on the degree of increase or decrease in total tumor burden compared to the baseline burden. The assessment was divided into the following five categories based on observation points: (1) complete remission (CR): complete disappearance of all lesions; (2) partial response (PR): a reduction in tumor burden of greater than or equal to 50% compared to baseline; (3) progressive disease (PD): compared with the baseline tumor burden, and the increase was greater than or equal to 25%; (4) stable disease (SD): no significant change in tumor size before and after chemotherapy; (5) shrink SD (SD-S): the tumor shrank slightly after chemotherapy but did not achieve PR.

The primary outcome was progression-free survival (PFS) defined as the time from first-time chemotherapy until the time of disease progression or death, which ever occurred first. To evaluate this endpoint, radiographic evaluations were conducted at follow-up intervals of every (6 ± 2) weeks during chemotherapy. Patients were followed up by outpatient and telephone after chemotherapy. The efficacy evaluation was determined by two or more physicians, at least one of whom held a deputy senior professional title or higher. Those assessing treatment response were blinded to the flow cytometry data.

### Immune infiltration analysis

The “correlation” module of the TIMER database (https://cistrome.shinyapps.io/timer/), was used to evaluate the correlation between CD28 and PD-1 in BC. The TISIDB database (cis.hku.hk/TISIDB/index.php) was used to analyze the correlation between CD28 and activated T cells and memory T cells.

### Statistical methods

The collection of 61 volunteers was convenience-based. Statistical analyses and graphical visualizations were performed using SPSS (v22.0), GraphPad Prism (v10.0), and R software (v4.4.2). Two-way ANOVA with multiple comparisons was used to analyze and compare expression levels of multiple immune cells and differences between three or more groups. We appled Bonferroni correction and reported adjusted p-values. Differences between the two groups were analyzed by unpaired or paired Student’s t test. Correlation analysis used the Spearman correlation coefficien. Receiver operating characteristic (ROC) curve analysis was performed in SPSS to evaluate the predictive value of the primary influencing factors of PD-1 and CD28 molecules on T cells for patient prognosis. Cut-off values were calculated using the maximum Youden index. The Kaplan-Meier survival curves with the Log-Rank test were used for comparing the PFS between groups using GraphPad Prism. Time-dependent ROC analysis was performed based on “timeROC”, package and visualized based on “ggplot2” package in R software. Univariate and multivariate Cox regression analyses were performed using the “rms” package and “ResourceSelection” package in R software. Subgroup analysis used “ordinal” package. P values (*p*) ≤ 0.05 were considered statistically significant. The Shapiro-Wilk test was used to evaluate the data. Before conducting univariate analysis, missing samples in the outcome column will be removed.

## Results

### Patient characteristics

Baseline characteristics of the 61 patients are summarised in Table 1. The mean age was 56 years (range 32–73 years). Based on the intrinsic phenotype, 26 patients had luminal, 21 had human epidermal growth factor receptor 2 (HER2)-enriched, and 14 had TNBC. All patients received standard 1st or 2nd line of chemotherapy.

**Table 1:** Patient characteristics.

Charateristics	n	%
Age, year, median (range)	56 (32-73)	
Gender		
female	61	100
male	0	0
Menopausal status		
Pre	15	24.59
Post	46	75.41
AJCC stage (TNM)		
Ⅲ	8	13.11
Ⅳ	53	86.89
Estrogen receptor		
positive	38	62.30
negative	23	37.70
Progesterone receptor		
positive	27	44.26
negative	34	55.74
HER-2/neu		
positive	19	31.15
negative	42	68.85
Surgery		
yes	34	55.74
no	27	44.26
Chemotherapy		
paclitaxel	8	13.11
TA	15	24.59
paclitaxel + Xeloda	14	22.95
THP	9	14.75
TP	9	14.75
TCbHP	5	8.20
other	1	1.64
lines of treatment		
1 line	52	85.25
2 line	8	14.75

TA: paclitaxel + anthracyclines; THP: paclitaxel + trastuzumab + pertuzumab; TP: paclitaxel + platinum; TCbHP: paclitaxel + carboplatin+ trastuzumab + pertuzumab.

**Fig 1:**
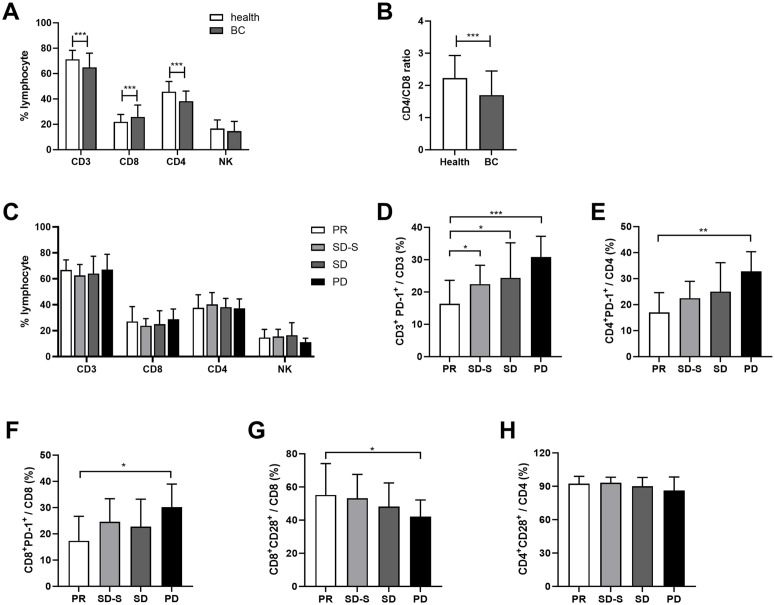
Expression frequency of T cell and their surface PD-1 and CD28. Distribution of T cells subsets, NK cells subsets (A) and CD4/CD8 ratio (B) in healthy volunteers and advanced BC patients was analyzed using t-test. C. Distribution of T cells and NK cells subsets in patients with PR, SD-S, SD and PD group. Expression of CD3^+^PD-1^+^ (D), CD4^+^PD-1^+^ (E), CD8^+^PD-1^+^ (F), CD8^+^CD28^+^ (G) and CD4^+^CD28^+^ (H) in patients with PR, SD-S, SD and PD group were analyzed. α = 0.05. Error bars indicate SD.

Selected 73 female health volunteers from health check-up as control group. Their breast ultrasound results showed no breast nodules or Breast Imaging Reporting and Data System (BI-RADS) categories 0−2. The mean age of the group was 56 years (range 32−73 years). Compared to the control group, patients with advanced BC had lower percentage of T cells, the CD4^+^ T helper cells (Ths) and CD4/CD8 ratio, while the proportion of CD8^+^ cytotoxic T lymphocytes (CTLs) increased in their peripheral blood (Fig 1A and B). There was no significant difference in the proportions of T cells, NK cells, CD3^+^PD-1^+^ cells, CD4^+^CD28^+^ cells and CD8^+^CD28^+^ cells among the Luminal, HER2-enriched and TNBC groups (S2 Fig).

### Patients exhibiting high CD8^+^CD28^+^ expression and low CD3^+^PD-1^+^ expression on peripheral T cells prior to paclitaxel chemotherapy demonstrated increased sensitivity to treatment

Three of the 61 patients who received chemotherapy did not complete the full course of treatment. Of those 55 patients, 11 were PD, 22 were SD, 12 were SD-S and 10 were PR. There was no significant difference in the proportions of T cells and NK cells among the four groups (Fig 1C). We further examined the expression levels of PD-1 and CD28 molecules on T cells further. We observed that increased sensitivity to chemotherapy was associated with lower PD-1 expression on CD3^+^ T cells (Fig 1D). The proportion of CD4^+^PD-1^+^ and CD8^+^PD-1^+^ cells was higher in the PD group than that in the PR group (Fig 1E and F). However, the proportion of cytotoxic CD8^+^CD28^+^ T cells were significantly higher in the PR group than that in the PD group (Fig 1G). There was no significant difference in the proportion of CD4^+^CD28^+^ T among the four groups (Fig 1H).

### CD3^+^PD-1^+^ and CD8^+^CD28^+^ expression on T cells can be used to predict clinical outcomes

**Fig 2:**
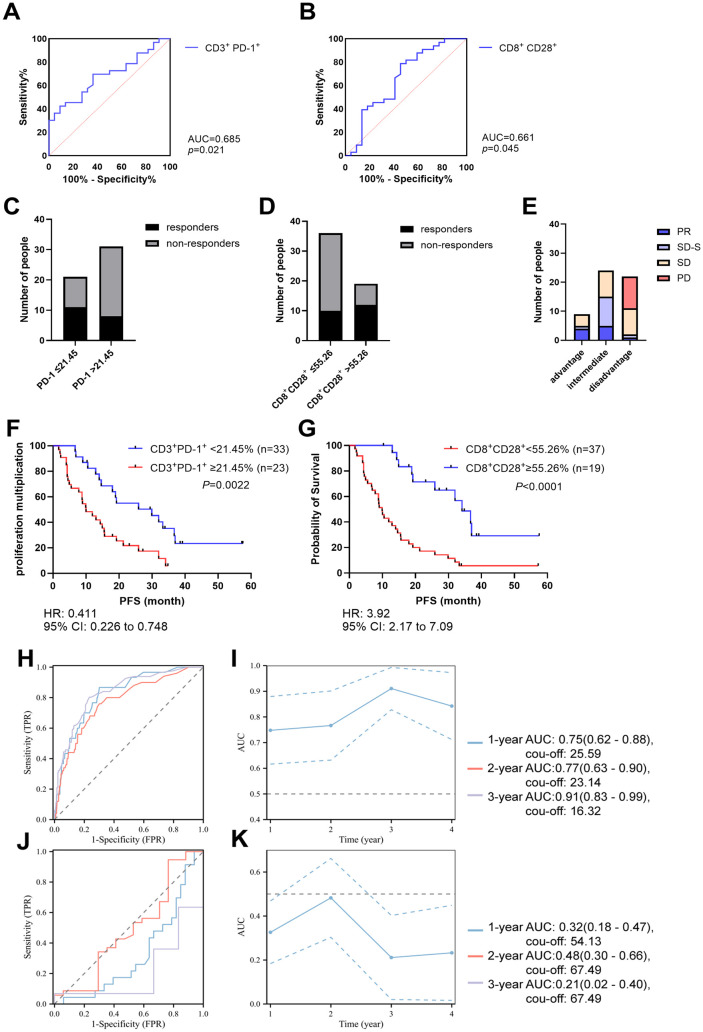
Diagnostic value of CD3+PD-1+ and CD8+CD28+ T cells expression in advanced BC. ROC curve analysis for CD3+PD-1+ (A) and CD8+CD28+ (B) T cells expression in advanced BC. Number of responders and non-responders in different expression levels of CD3^+^PD-1^+^ (C) and CD8^+^CD28^+^ (D). Kaplan-Meier curve for PFS classified by different expression level of CD3^+^PD-1^+^ (F) and CD8^+^CD28^+^ (G) T cells in advanced BC. Time-dependent ROC analysis of CD3^+^PD-1^+^ (H) and CD8^+^CD28^+^ (J). Time-dependent AUC analysis of CD3^+^PD-1^+^ (I) and CD8^+^CD28^+^ (K).

Patients were divided into responders group (PR and SD-S) and non-responders group (SD and PD). ROC subject curve was used to determine the diagnostic role and cut-off values of CD3^+^PD-1^+^ and CD8^+^CD28^+^ in two groups. Area under the curve (AUC) was 0.685 (95% confidence internal [CI] 0.547–0.824) and 0.661 (95% CI 0.505–0.816), with cut-off values of 21.45% and 55.27%, respectively (Fig 2A and 2B). Upon observing the responses of patients to treatment above and below the critical values, it was found that the majority of non-responsive patients were in the queue above 21.45% and below 55.27% (Fig 2C and 2D). To improve the accuracy of assessing the clinical therapeutic effects of CD3^+^PD-1^+^ and CD8^+^CD28^+^, we defined the following groups: Advantage: CD3^+^PD-1^+^<21.45% and CD8^+^CD28^+^>55.27%, Intermediate: CD3^+^PD-1^+^<21.45% or CD8^+^CD28^+^>55.27%, Disadvantage: CD3^+^PD-1^+^>21.45% and CD8^+^CD28^+^<55.27%. The vast majority of PR and SD-S patients were in the advantage and intermediate groups, while all PD patients were all in the disadvantage group (Fig 2E).

Univariate and multivariate Cox analysis revealed that year, intrinsic subtypes, stage, chemotherapy protocol and menopausal status were not independent factors influencing CD3^+^PD-1^+^ expression in advanced BC patients. Only the chemotherapy protocol was an independent factor influencing of CD8^+^CD28^+^ expression (Table 2). We conducted a comparative analysis of the treatment response in conventional models and new models (Table 3). Of the 61 enrolled patients, 58 were eligible for clinical efficacy evaluation and underwent net reclassification index (NRI) and integrated discrimination improvement (IDI) analyses. The NRI for the new models was 59.31% (95% CI 10.14%−108.48%; *p* = 0.018), and the IDI was 0.084 (95% CI 0.011–0.157, *p* = 0.024). These results provide substantial evidence supporting the assertion that the incremental effect of CD3^+^PD-1^+^ and CD8^+^CD28^+^ on the predictive value of treatment response was greater than that of the conventional models.

**Table 2:** Univariate and multivariate analysis of CD3^+^PD-1^+^ and CD8^+^CD28^+^ in BC.

Characteristics		CD3 ^+^ PD-1^+^				CD8 ^+^ CD28^+^			
	Total(N)	OR (95% CI) Univariate analysis	P value	OR (95% CI) Multivariate analysis	P value	OR (95% CI) Univariate analysis	P value	OR (95% CI) Multivariate analysis	P value
**Age, y**	61	1.015 (0.965–1.067)	0.568	1.002 (0.930–1.078)	0.966	0.967 (0.919–1.019)	0.208	0.971 (0.900–1.049)	0.456
**Intrinsic subtypes**	61								
luminal	26	Reference		Reference		Reference		Reference	
Her2-enriched	21	0.978 (0.306–3.128)	0.97	0.859 (0.134–5.524)	0.873	0.467 (0.138–1.582)	0.221	0.115 (0.011–1.230)	0.074
TNBC	14	0.550 (0.148–2.046)	0.372	0.735 (0.103–5.256)	0.759	1.556 (0.420–5.763)	0.508	0.916 (0.113–7.405)	0.934
**Stages**	61								
Ⅲ	8	Reference		Reference		Reference		Reference	
Ⅳ	53	2.174 (0.470–10.049)	0.32	3.414 (0.212–55.078)	0.387	0.202 (0.037–1.099)	0.064	0.000 (0.000 – Inf)	0.99
**Chemotherapy protocol**	60								
paclitaxel	8	Reference		Reference		Reference		Reference	
Paclitaxel + Xeloda	14	2.500 (0.410–15.230)	0.32	2.364 (0.237–23.563)	0.463	1.667 (0.240–11.575)	0.605	6.673 (0.494–90.041)	0.153
TA	15	1.143 (0.205–6.366)	0.879	3.327 (0.239–46.268)	0.371	3.429 (0.516–22.802)	0.202	1.361 (0.051–36.618)	0.854
TCbHP	5	0.667 (0.069–6.409)	0.725	0.133 (0.005–3.242)	0.216	2.000 (0.181–22.056)	0.571	28.257 (0.667–1197.376)	0.08
THP	9	1.250 (0.185–8.444)	0.819	0.582 (0.040–8.361)	0.69	3.750 (0.473–29.752)	0.211	53.068 (1.645–1711.591)	**0.025**
TP	9	0.800 (0.118–5.404)	0.819	0.980 (0.018–53.162)	0.992	2.400 (0.303–19.041)	0.407	1.582 (0.020–124.713)	0.837
**Menopausal status**	61								
Pre	15	Reference		Reference		Reference		Reference	
Post	46	2.132 (0.650–6.993)	0.212	1.488 (0.050–44.599)	0.819	0.804 (0.249–2.596)	0.716	0.294 (0.010–9.072)	0.484

Univariate and multivariate Cox proportional hazard regressionmodels were performed to identify associations with CD3^+^PD-1^+^ and CD8^+^CD28^+^. Logistic regression was performed using the cut-off values for PD-1 and CD28. TA: paclitaxel + anthracyclines; THP: paclitaxel + trastuzumab + pertuzumab; TP: paclitaxel + platinum; TCbHP: paclitaxel + carboplatin+ trastuzumab + pertuzumab. Bold values indicate statistical significance. OR, odds ratio. CI, confidence interval.

**Table 3:** Comparisons of capacity between conventional models and multivariable models.

Variables	NRI			IDI		
	Estimate	95 CI	P value	Estimate	95 CI	P value
Conventional models	Ref.			Ref.		
New models	59.31%	10.147% ~ 108.481%	0.018	0.084	0.011 ~ 0.157	0.024

Conventional models: include year, intrinsic subtypes, stages and Menopausal status. New models: conventional models plus CD3^+^PD-1^+^ and CD8^+^CD28^+^. NRI: net reclassification improvement; IDI: integrated discrimination improvement.

Survival analysis revealed that patients with low CD3^+^PD-1^+^ expression (HR 0.411, 95% CI 0.226–0.748, *p* = 0.0022) and high CD8^+^CD28^+^ expression (HR 3.92, 95% CI 2.17–7.09, *p* < 0.0001) had longer PFS (Fig 2F and G). Time-dependent ROC analysis of CD3^+^PD-1^+^ and CD8^+^CD28^+^ was performed. The results showed that the AUCs and the cut-off values for CD3^+^PD-1^+^ were 0.75 (95% CI 0.62–0.88, 25.59), 0.77 (95% CI 0.63–0.90, 23.14) and 0.91 (95% CI 0.83–0.99, 16.32) for 1-, 2- and 3-year PFS, respectively (Fig. 2H and I). The AUCs and the cut-offs values for CD8^+^CD28^+^ were 0.32 (95% CI 0.18–0.47, 54.13), 0.48 (95% CI 0.35–0.65, 67.49) and 0.21 (95% CI 0.17–0.83, 67.49) for 1-, 2- and 3-year PFS, respectively (Fig. 2J and K). These results indicated that the CD3^+^PD-1^+^ and CD8^+^CD28^+^ to have valid predictive value for PFS in advanced breast cancer. The obtained cut-offs were also close to those in the above model.

A statistically significant association was observed between CD3^+^PD-1^+^ level and PFS in over 60 years old (95% CI 1.832–38.912, *p* = 0.022), luminal (95% CI 1.394–13.497, *p* = 0.011), stages Ⅳ (95% CI 1.596–5.790, *p* = 0.001), paclitaxel + Xeloda (95% CI 1.012–69.299, *p* = 0.049), pre-menopausal (95% CI 1.619–127.557, *p* = 0.017) and post-menopausal (95% CI 1.193–4.935, *p* = 0.014) subgroups. A statistically significant difference in CD8^+^CD28^+^ levels was observed in TNBC (95% CI 0.031–0.843, *p* = 0.031), TA (95% CI 0.011–0.780, *p* = 0.029), and post-menopausal (95% CI 0.244–0.975, *p* = 0.042) subgroups (Table 4). PD-1 (β coefficient −0.701, p = 0.009) were associated with a shorter PFS, whereas CD28 was negatively associated with PFS (β coefficient 0.192, *p* = 0.731) in group TA.

**Table 4:** Subgroup analysis for the associations between CD3^+^PD-1^+^/ CD8^+^CD28^+^ and PFS.

Variables	CD3 ^+^ PD-1^+^		CD8 ^+^ CD28^+^	
	HR (95% CI)	P value	HR (95% CI)	P value
**Age, y**				
>60	8.443 (1.832 - 38.912)	**0.006**	0.563 (0.192 - 1.648)	0.294
≤60	2.128 (0.997 - 4.544)	0.051	0.497 (0.229 - 1.077)	0.076
**Intrinsic subtypes**				
luminal	4.337 (1.394 - 13.497)	**0.011**	0.753 (0.301 - 1.882)	0.544
Her2-enriched	1.910 (0.723 - 5.044)	0.191	1.317 (0.438 - 3.961)	0.624
TNBC	4.632 (0.800 - 26.825)	0.087	0.161 (0.031 - 0.843)	**0.031**
**Stages**				
Ⅲ	–	–	–	–
Ⅳ	3.039 (1.596 - 5.790)	**0.001**	0.794 (0.424 - 1.487)	0.471
**Chemotherapy protocol**				
paclitaxel	2.547 (0.460 - 14.092)	0.284	0.714 (0.073 - 6.965)	0.772
Paclitaxel + Xeloda	8.376 (1.012 - 69.299)	**0.049**	0.637 (0.190 - 2.132)	0.464
TA	4.296 (0.797 - 23.161)	0.09	0.091 (0.011 - 0.780)	**0.029**
THP	3.581 (0.673 - 19.058)	0.135	0.750 (0.161 - 3.499)	0.715
TP	6.855 (0.746 - 62.943)	0.089	0.997 (0.197 - 5.055)	0.997
TCbHP	0.879 (0.075 - 10.261)	0.918	0.390 (0.034 - 4.438)	0.448
other	–	–	–	–
**Menopausal status**				
Pre	14.373 (1.619 - 127.557)	**0.017**	0.802 (0.188 - 3.424)	0.766
Post	2.426 (1.193 - 4.935)	**0.014**	0.487 (0.244 - 0.975)	**0.042**

The models were adjusted for age, intrinsic subtypes, stages, chemotherapy protocol and menopausal status.

TA: paclitaxel + anthracyclines; THP: paclitaxel + trastuzumab + pertuzumab; TP: paclitaxel + platinum; TCbHP: paclitaxel + carboplatin+ trastuzumab + pertuzumab. Bold values indicate statistical significance. HR, Hazard Ratio.

### Blocking PD-1 enhances the ability of PBMCs to kill tumor cells and is closely related to CD8^+^CD28^+^ level

**Fig 3:**
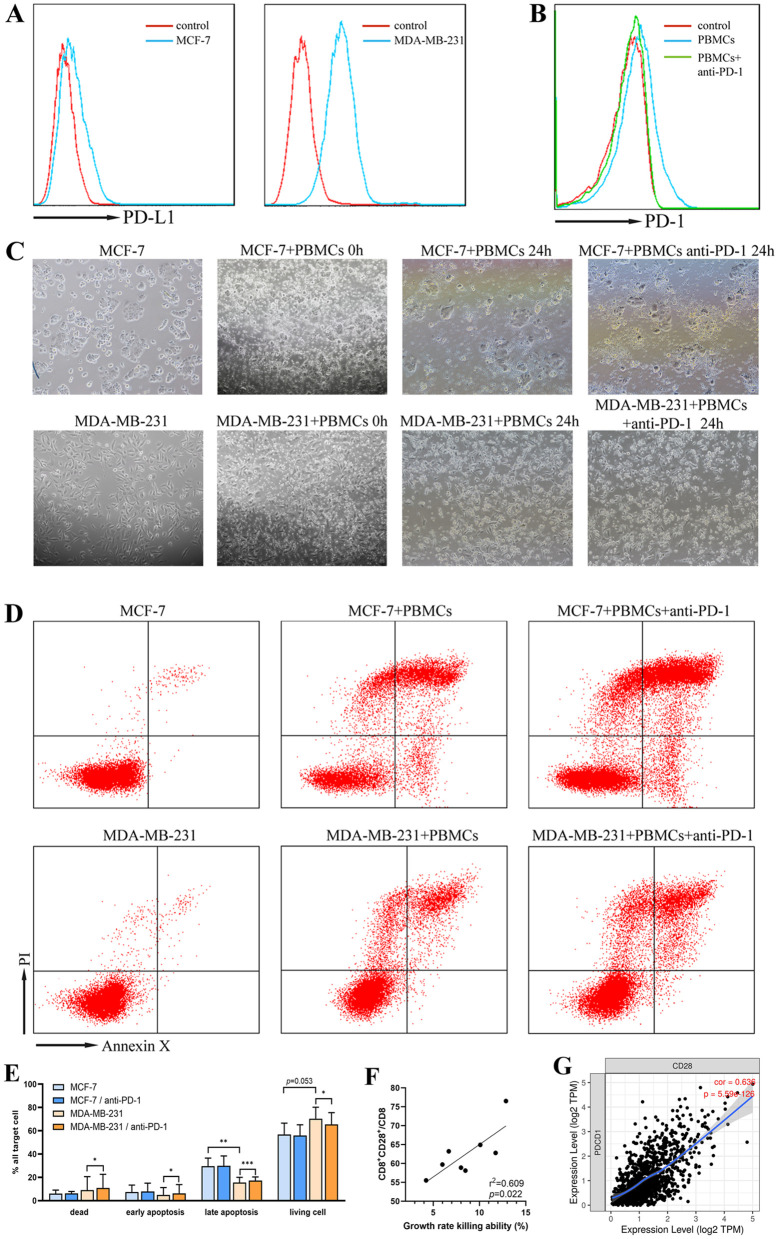
Analysis of killing parameters of MCF-7 and MDA-MB-231 cells by PBMCs. A. Expression of PD-L1 on MCF-7 and MDA-MB-231 cells. B. Blocking effect of anti-PD-1 antibody on PBMCs. C. Pictures of PBMCs co-cultured with MCF-7 or MDA-MB-231 cells. D & E. Cytotoxic effects of PBMCs alone and with anti-PD-1 antibody, on MCF-7 or MDA-MB-231 cells following 24h incubation was analyzed. F. The correlation between the enhanced killing effect of PBMCs on MDA-MB-231 cells by anti-PD-1 antibody and expression of CD8^+^CD28^+^ on CD8^+^ T cells was analyzed using correlation. G. The correlated between PD-1 and CD28 molecules in BC were associated with multiple immune cell infiltration in TIMER database. α = 0.05. Error bars indicate SD.

MDA-MB-231 cells had higher PD-L1 expression on their surface than MCF-7 cells (Fig 3A). No expression of PD-1 molecule was detected in PBMCs treated with anti-PD-1 antibodies compared to the control group (Fig 3B). PBMCs were cultured with MCF-7 or MDA-MB-231 cells for 24h. The PBMCs migrated toward and surrounded the tumor cells (Fig 3C). PBMC-mediated killing was more effective against MCF-7 cells than against MDA-MB-231 cells. However, after adding anti-PD-1 antibody, the proportion of cell death and apoptosis increased in MDA-MB-231 cells with high PD-L1 expression (Fig 3D and E). The enhanced killing effect of PBMCs on MDA-MB-231 cells by anti-PD-1 antibody was positively correlated with CD8^+^CD28^+^ expression on PBMCs (Fig 3F). The TIMER database showed that PD-1 gene (Sfig 3) and CD28 gene (S4 Fig) in BC were associated with multiple immune cell infiltration. There is a correlation between them in tumor microenvironment of breast cancer (Fig 3G).

### Blocking PD-1 on PMBCs can induce them to produce tumor-specific effector memory T cells

**Fig 4:**
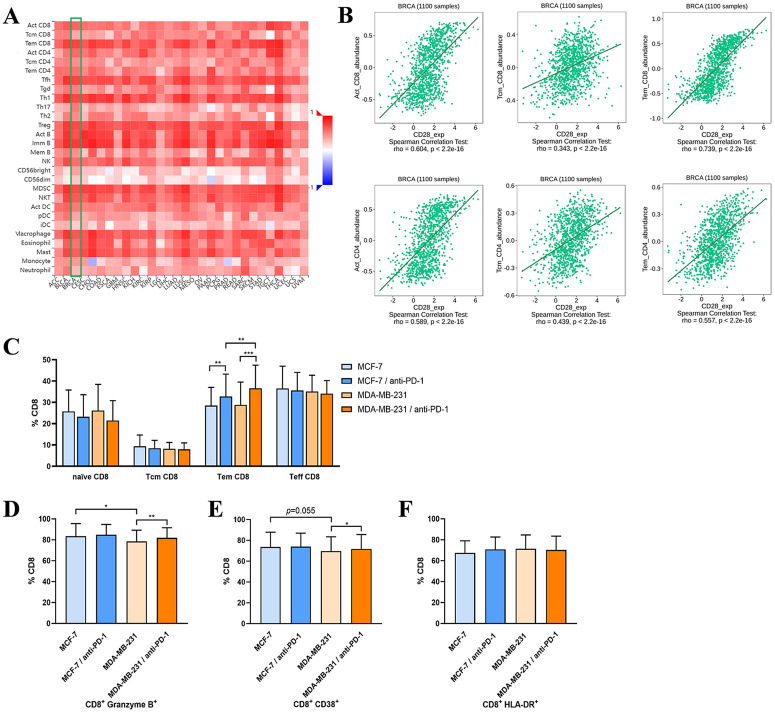
Correlation analysis between CD28 and lymphocytes. A. Heatmap analysis of the correlation between CD28 and lymphocytes in tumors. B. CD28 in BC is positively correlated with activated, Tcm and Tem cells on CD8+ T and CD4^+^T cells. C. The expression of naive, Tcm, Tem and Teff on CD8^+^ T cells in PBMCs alone and with anti-PD-1 antibody co-cultured with MCF-7 or MDA‐MB‐231 cells. The expression of granzyme B (D), CD38 (E) and HLA-DR (F) on CD8^+^ T cells in PBMCs alone or with anti-PD-1 antibody co-cultured with MCF-7 or MDA-MB-231 cells following 24h incubation. α = 0.05. Error bars indicate SD.

The TISIDB database analysis showed that CD28 expression in BC tumor was associated with the abundance of activated CD8^+^ T cells, CD8^+^ T central memory (Tcm) cells and CD8^+^ T effective memory (Tem) cells, particularly CD8^+^ Tem cells (Fig 4A and B). In vitro experimental results showed that the proportion of CD8^+^ Tem cells in PBMCs treated with PD-1 antibody increased after co-culturing with MCF-7 and MDA-MB-231 cells, respectively. In the MDA-MB-231 cells group with a significant killing effect, the proportion of CD8^+^ Tem cells increased more than that in the MCF-7 cells group (Fig 4C). Compared to MDA-MB-231 cells, PBMCs co-cultured with MCF-7 cells secreted more GzmB and showed increased CD38 expression in CD8^+^ T cells. PD-1 blockade upregulated GzmB and CD38 expression in MDA-MB-231 cultures (Fig 4D and E). However, there was no change in HLA-DR expression on CD8^+^ T cells (Fig 4F). Those results suggest that binding of PD-L1 on tumor cells to PD-1 on T cells affects T cells activation and Tem cells production.

## Discussion

T cells represent a major cellular subset responsible for mediating specific anti-tumor immunity. T cell activation requires an initial signal from the TCR and a second signal from the CD28 molecule [14]. CD28 is a major costimulatory receptor that regulates T cell proliferation, survival and effector functions. Downregulation of the CD28 molecule is considered a manifestation of T cell senescence or exhaustion. This is characterized by IL-2, IFN-γ, tumor necrosis factor alpha (TNF-α), and GzmB secretion decreased, inhibiting TCR-dependent antigen-specific killing [15,16]. The peripheral frequencies of CD8 ⁺ CD28⁺ and CD8⁺CD28⁻ T cells served as prognostic biomarkers in NSCLC patients treated with chemo(radio)therapy; those with a high CD8 ⁺ CD28 ⁺ T cell count and/or a low CD8⁺CD28⁻ T cell count predicted significantly better OS and PFS [17,18]. In lung adenocarcinoma, elevated CD28 expression within the tumor immune microenvironment (TIME) is not only correlated with poorer disease-free survival (DFS), but also linked to increased abundance and robust activation of lymphocytes, suggesting a potential role in modulating TIME [19]. PD-1 is a co-inhibitory receptor that can reduce the anti-tumor effect of immune cells and promote tumor cell proliferation by inhibiting T cell activation. In our study, patients with favorable outcomes exhibited lower PD-1 and high CD28 expression. BC patients with a favorable response to chemotherapy and prolonged PFS exhibited lower CD3^+^PD-1^+^ and higher CD8^+^CD28^+^ expression on peripheral blood T cells.

Recent studies have reported that the CD28 receptor is the main target of anti-PD-1/PD-L1 therapy [20,21]. Ki67-expressing CD8^+^ T cells are increase in blood of approximately half of lung cancer patients after anti-PD-1 therapy. These CD8^+^ T cells, which are activated by PD-1 treatment mainly CD28^+^ [22]. Murine model research results show that after treatment with co-expression plasmid of CD28-siRNA-PD-1 mice, PD-1 expression in tumor tissue was inhibited while CD28 expression was significantly increased [23]. *In vitro* experiments demonstrate that PBMCs exhibit weaker cytotoxicity against PD-L1-high MDA-MB-231 cells than against PD-L1-low MCF-7 cells. Blocking PD-1 can potentiate the cytotoxic activity of PBMCs against MDA-MB-231 cells, and the degree of enhancement correlates with CD28 expression on CD8^+^ T cells. In mouse colon cancer model, 8 of 10 mice implanted with high expression of CD86 (ligand of the CD28 molecule) on MC38 cells became tumor-free after anti-PD-1 treatment, whereas 1 of 10 mice implanted with control MC38 cells did so. Mice with high CD86 expression could resist tumor rechallenge for over 60 days without additional treatment [24]. Those results suggest that the reduced anti-tumor effect of T cells with high PD-1 expression is related to CD28 receptor expression on their surface. CD28 may represent a useful biomarker for PD-1 immune checkpoint blockade (ICB) in breast cancer. Regarding soluble PD-1, one study showed that its levels remained unchanged before and after neoadjuvant chemotherapy in BC [25]. Furthermore, no statistically significant associations were established between sPD-1 levels and clinical stage, individual TNM system criteria, tumor histological structure, grade, receptor status, or molecular type [26].

The generation and persistence of memory T cells are critical components of anti-tumor immunity due to their prolonged persistence, rapid activation, and migration. when T cells and antigens meet again, Tcm cells can self-renew and differentiate into Tem cells, effector T (Teff) cells, and tissue-resident memory T (Trm) cells. Tcm cells have lymphoid homing properties and high a proliferation ability, and Tem cells produce more effector cytokines [27]. In hepatocellular carcinoma patients with durable responses to anti-PD-L1 combined antibody and bevacizumab, tumors are rich in PD-L1^+^CXCL10^+^ macrophages that can recruit peripheral CD8 Tem cells into the tumor. These CD8 Tem cells differentiate preferentially into PD-1^-^CD45RA^+^CD8^+^ Tem cells with potent cytotoxicity [28]. In addition, Huang et al. identified the presence of tumor antigen-specific memory CD8^+^ T cells in tumor draining lymph nodes and confirmed the group of cells’ critical role in PD-1 ICB [29]. In metastatic melanoma patients who had significant clinical response to immunotherapy, the therapy immunotherapy induced memory T cells, including Trms in tumors and skin and Tem in the blood. These memory cells can persist for up to 9 years [30]. This study also observed similar results, reducing or blocking PD-1 expression can induce Tem cells, which can provide long-term protection for cancer patients. Data analysis revealed that CD28 expression is associated with the infiltration of multiple immune cells in the TIME, including activated T cells and memory T cells. Blocking PD-1 increased the proportion of Tem in PBMCs increased, even when co-cultured with MCF-7 cells. Additionally, blocking PD-1 enhanced T cells activation through the upregulation of CD38 expression and GzmB secretion.

Unlike circulating memory T cells (such as Tcm and Tem cells), Tem cells persist long-term in non-lymphoid tissues rather than recirculating in peripheral blood. A study found that patients with a higher proportion of CD103^+^CD8^+^ T cells in tumor infiltrating lymphocytes (TILs) have a longer overall survival rate in a variety of cancer types, such as lung cancer, BC, colorectal cancer. There is phenotypic overlap between Trm cells and T cell exhaustion (Tex), such as PD-1 [31]. This makes it difficult to determine if these T cells still have anti-tumor function or have entered a state of functional exhaustion. Furthermore, it is controversial whether T cells with a Trm phenotype in tumor tissues are bona fide tissue-resident populations or are simply circulating T cells in a transient resting state. Therefore, it is recommended that PD-1 be detected simultaneously on peripheral blood and Trm cells.

Although chemotherapy leads to increased infiltration of CD8^+^ T cells in tumor microenvironment, but these cells exhibit a state of exhaustion [32]. T cell exhaustion is characterized by the expression of PD-1, TIM-3, and LAG3. In ovarian cancer models, inhibiting the expression of PD-L1 and CD80 (CD28 ligand) in tumor cells improves the efficacy of chemotherapy [33]. Currently, PD-1 ICB studies in BC mainly focus on TNBC, and the therapeutic effectiveness is limited. In the KEYNOTE086 study, the overall response rate (ORR) for 170 advanced TNBC patients was only 5.3%. Patients with PD-L1 positive expression did not show a significant improvement in response rate, with an ORR is only 5.7% [31]. In the Impassion130 trial, patients who received PD-1 ICB in combination with paclitaxel (nP) had longer PFS than patients who received nP alone first-line immunotherapy for advanced TNBC. Subgroup analysis shows a more significant improvement in PD-L1 positive patients. The percentages of CD8^+^ T cells and immune cells expressing PD-L1, were moderately correlated when evaluated as continuous variables [34,35]. The patient population exhibited prolonged PFS when treated with the chemotherapy regimen, regardless of whether immunotherapy was added. Currently, the primary biomarker used to predict response to PD-1/PD-L1 ICB is immunohistochemical (IHC) detection of PD-L1 expression in tumor tissues. The level of expression is used to assess the impact of tumor cells on immune cells within the tumor microenvironment. Our study predicts paclitaxel chemotherapy response by evaluating the activation status of lymphocytes, as measured by the expression levels of CD3^+^PD-1^+^ (exhausted T cells) and CD8^+^CD28^+^ (cytotoxic T cells). Therefore, they may serve not only as biomarkers to evaluate the efficacy of paclitaxel combined with immunotherapy or immunotherapy alone in TNBC, but could also other BC subtypes. However, it is unclear whether they can be generalized to evaluate other chemotherapy regimens. Further research is required to validate this.

Our research had several limitations. First, the number of participants was limited. This exploratory study used convenience sampling, and findings should therefore be interpreted as hypothesis-generating. Second, since we included all patients with advanced breast cancer who received paclitaxel based first- or second-line chemotherapy, the treatment plans were inconsistent. Larger and more representative cohort studies are required to confirm the survival and clinical relevance of these findings across in chemotherapy regimens and subgroups, even whether simple PD-1 and CD28 flow cytometry analysis can be incorporated into the Artificial Bee Colony Algorithm （ABC） management algorithm. Third, our mechanistic investigation was confined to in vitro systems. Fourth, information on the PD-L1 expression in BC patients’ tumor was unavailable. Subsequent studies will use in vivo models determine the roles and mechanisms of CD28 and PD-1 in anti-tumor immunity. Despite these limitations, to the best of our knowledge, this is the first study to identify pretreatment CD28 and PD-1 levels in peripheral blood as potential indicators of chemotherapy response and survival in advanced BC patients. Advantages include easy to obtain materials, simple and economical detection methods, and dynamic observation.

In conclusion, our study suggests that pretreatment the expression of PD-1 and CD28 molecules on the surface of T cells in peripheral blood may serve as potential biomarkers for assessing tumor progression, chemotherapy response and prognosis in advanced cancer patients. In vitro experiments indicate that in the process of anti-tumor immune response in patients with high expression of PD-L1, blocking PD-1 expression can improve tumor killing effect of PBMCs (this process is closely related to the expression of CD28 molecules) and induce more memory and activated T cells, providing long-term protection for preventing tumor progression or recurrence. Additionally, the results of MCF-7 model with low PD-L1 expression showed that, even if PD-1 ICB treatment was ineffective, more memory T cells and could still be induced. Those results are expected to inform the treatment of BC with PD-1 ICB.

## Supporting information

S1 Fig Gating strategy of Flow-Cytometry. Dot plots showing the expression of the PD-1^+^ and CD28^+^ marker on CD3, CD4 and CD8 T cells in peripheral blood (A). Dot plots showing the expression of the naive T cells, Tcm, Tem, Teff, Gzm-B, CD38 and HLA-DR on CD8 in PBMCs (B).
(TIF)

S2 Fig Expression frequency of T cells and their surface markers PD-1 and CD28 across different intrinsic subtypes. Distribution of T cells subsets and NK cells subsets (A), CD3^+^PD-1^+^ (B), CD4^+^CD28^+^ (C) and CD8^+^CD28^+^ (D) among Luminal, Her2-enriched and TNBC groups. Error bars indicate SD.
(TIF)

S3 Fig Associations between PD-1 expression levels and B cells, CD4+ T cells, CD8+ T cells, macrophage, neutrophil and dendritic cells counts based on TIMER in Breast Invasive Carcinoma (BRCA), BRCA-Basal, BRCA-Her2 and BRCA-Luminal. (TIF)

S4 Fig Associations between CD28 expression levels and B cells, CD4+ T cells, CD8+ T cells, macrophage, neutrophil and dendritic cells counts based on TIMER in Breast Invasive Carcinoma (BRCA), BRCA-Basal, BRCA-Her2 and BRCA-Luminal. (TIF)

S1 File This is the file including all data underlying the findings described in this manuscript. (XLSX)
