# Supplementary figures and images for: The PD-1 and CD28 molecules on T cells in peripheral blood are associated with the prognosis of patients with advanced breast cancer receiving paclitaxel chemotherapy
Source: PLoS One. 2026 Apr 6;21(4):e0344366. doi: 10.1371/journal.pone.0344366 (PMC13052852; doi:10.1371/journal.pone.0344366)

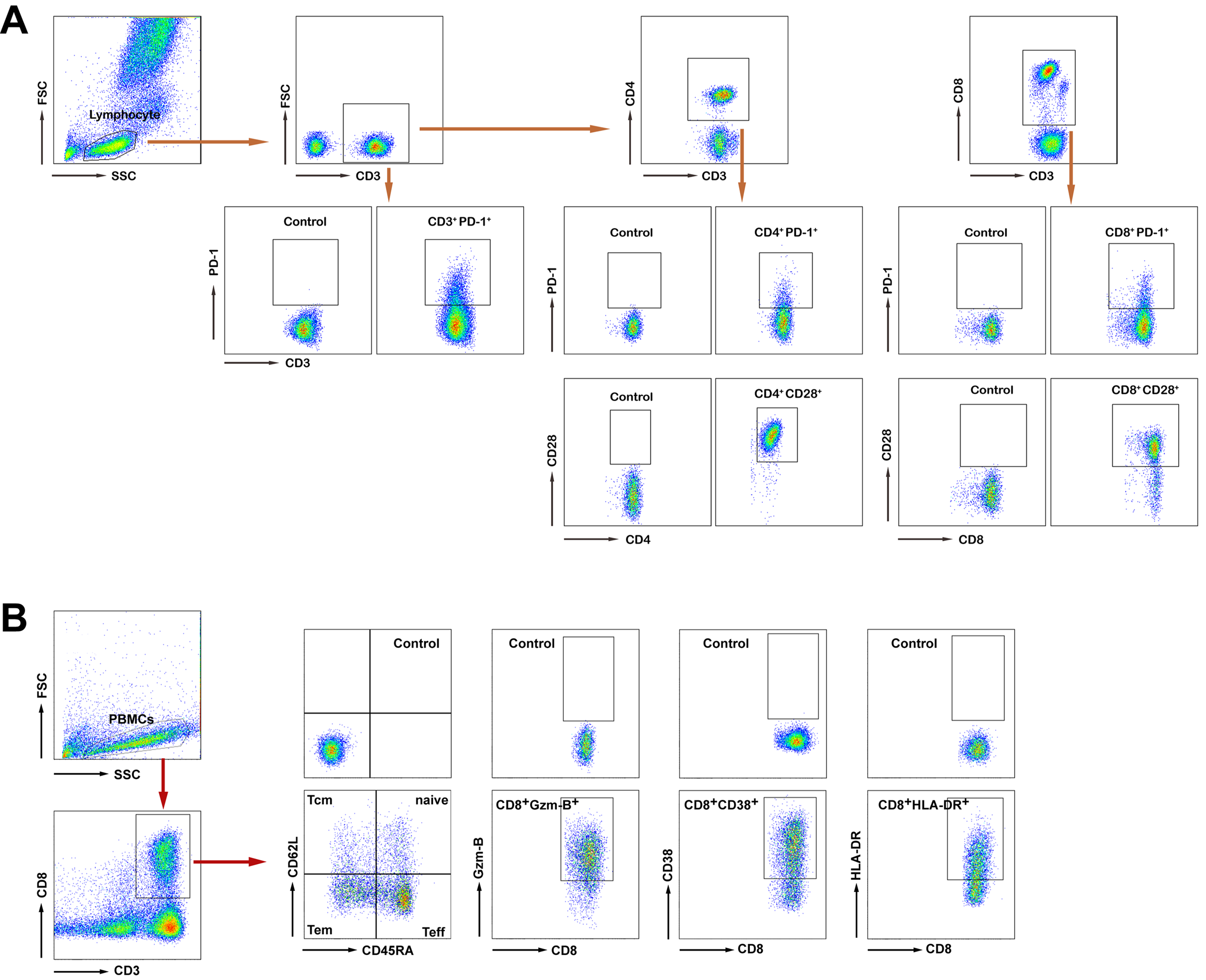

Supplement: S1 Fig — Dot plots showing the expression of the PD-1+ and CD28+ marker on CD3, CD4 and CD8 T cells in peripheral blood (A). Dot plots showing the expression of the naive T cells, Tcm, Tem, Teff, Gzm-B, CD38 and HLA-DR on CD8 in PBMCs (B). (TIF) [file pone.0344366.s001.tif]

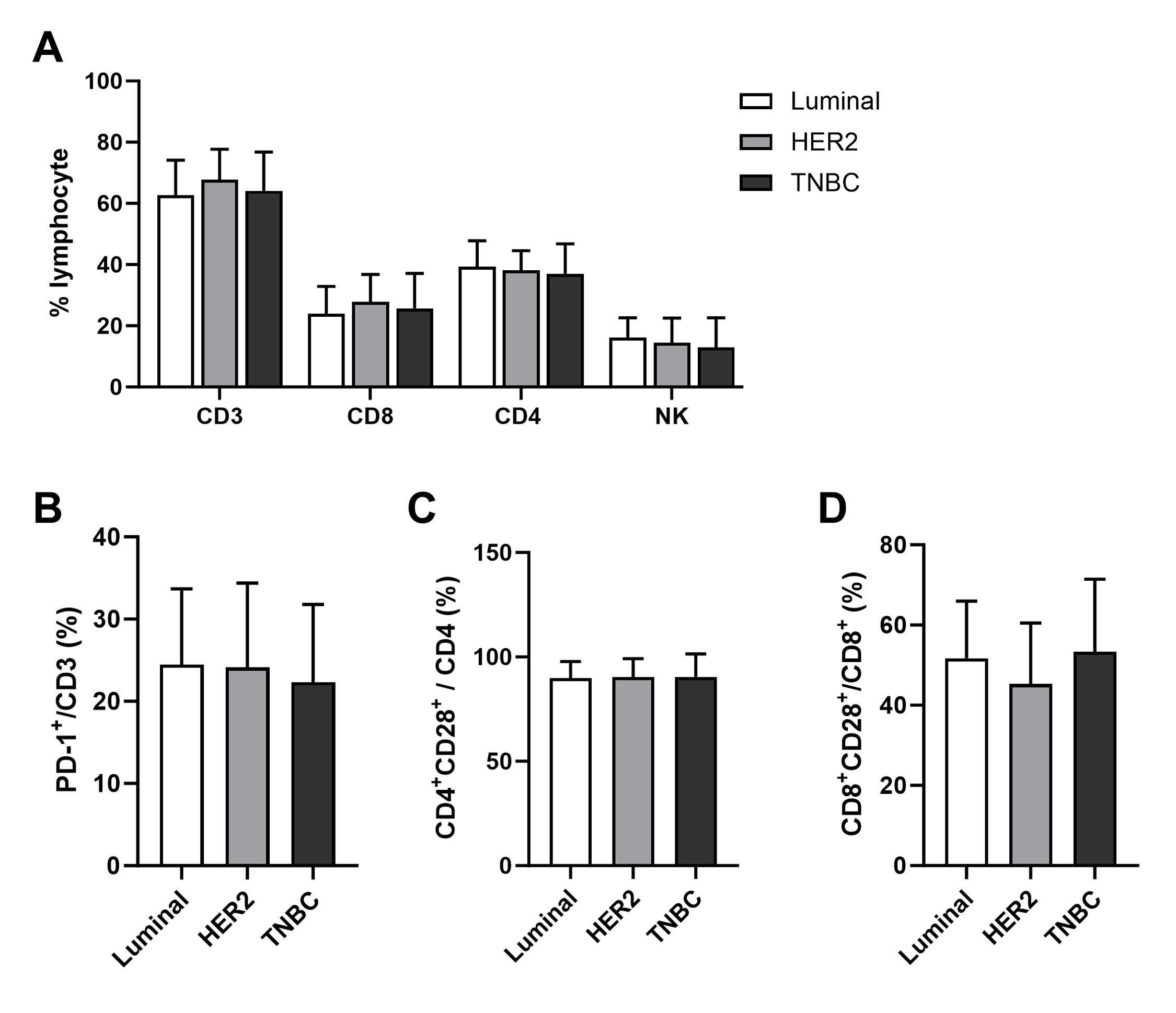

Supplement: S2 Fig — Distribution of T cells subsets and NK cells subsets (A), CD3+PD-1+ (B), CD4+CD28+ (C) and CD8+CD28+ (D) among Luminal, Her2-enriched and TNBC groups. Error bars indicate SD. (TIF) [file pone.0344366.s002.tif]

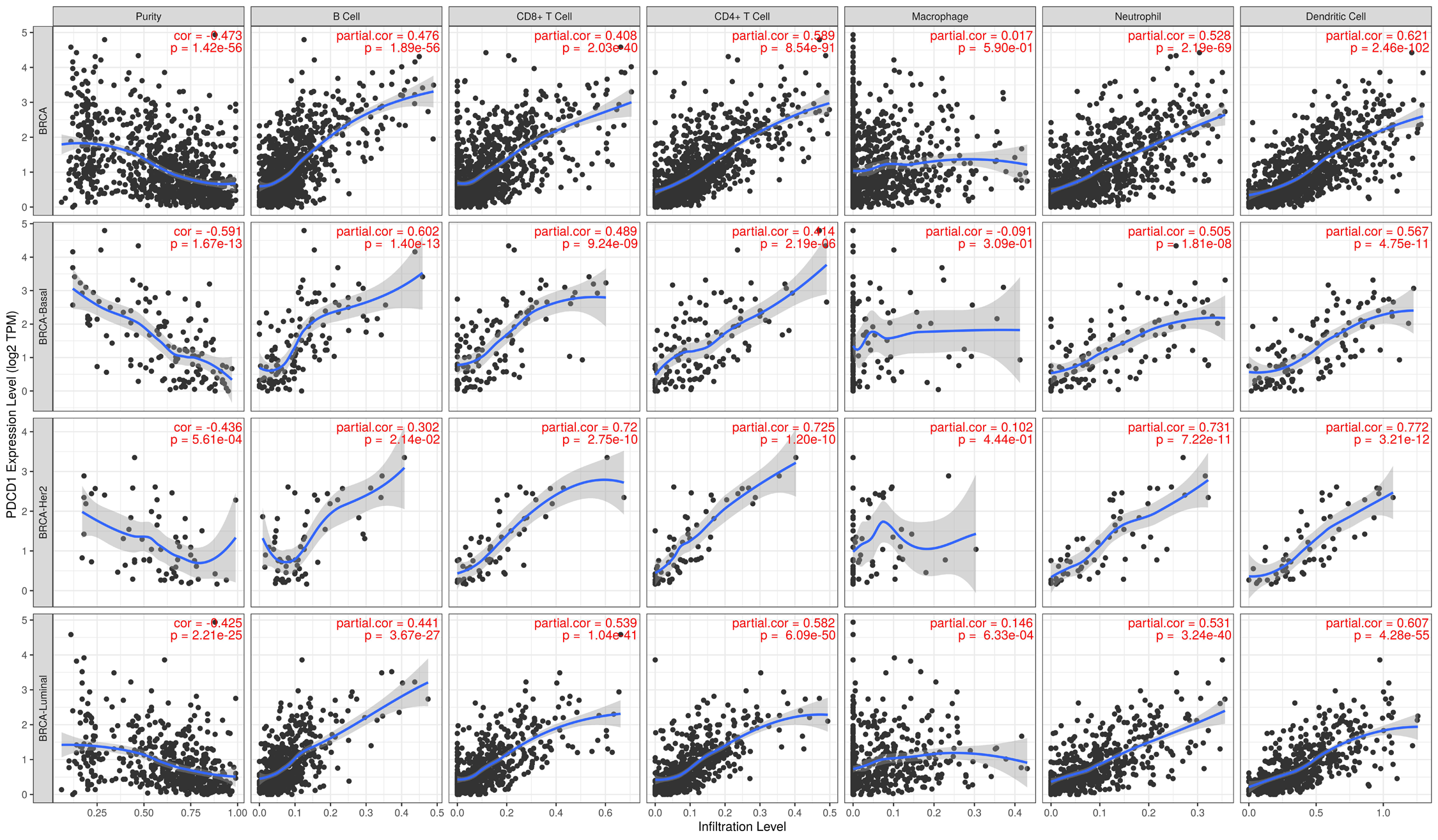

Supplement: S3 Fig — (TIF) [file pone.0344366.s003.tif]

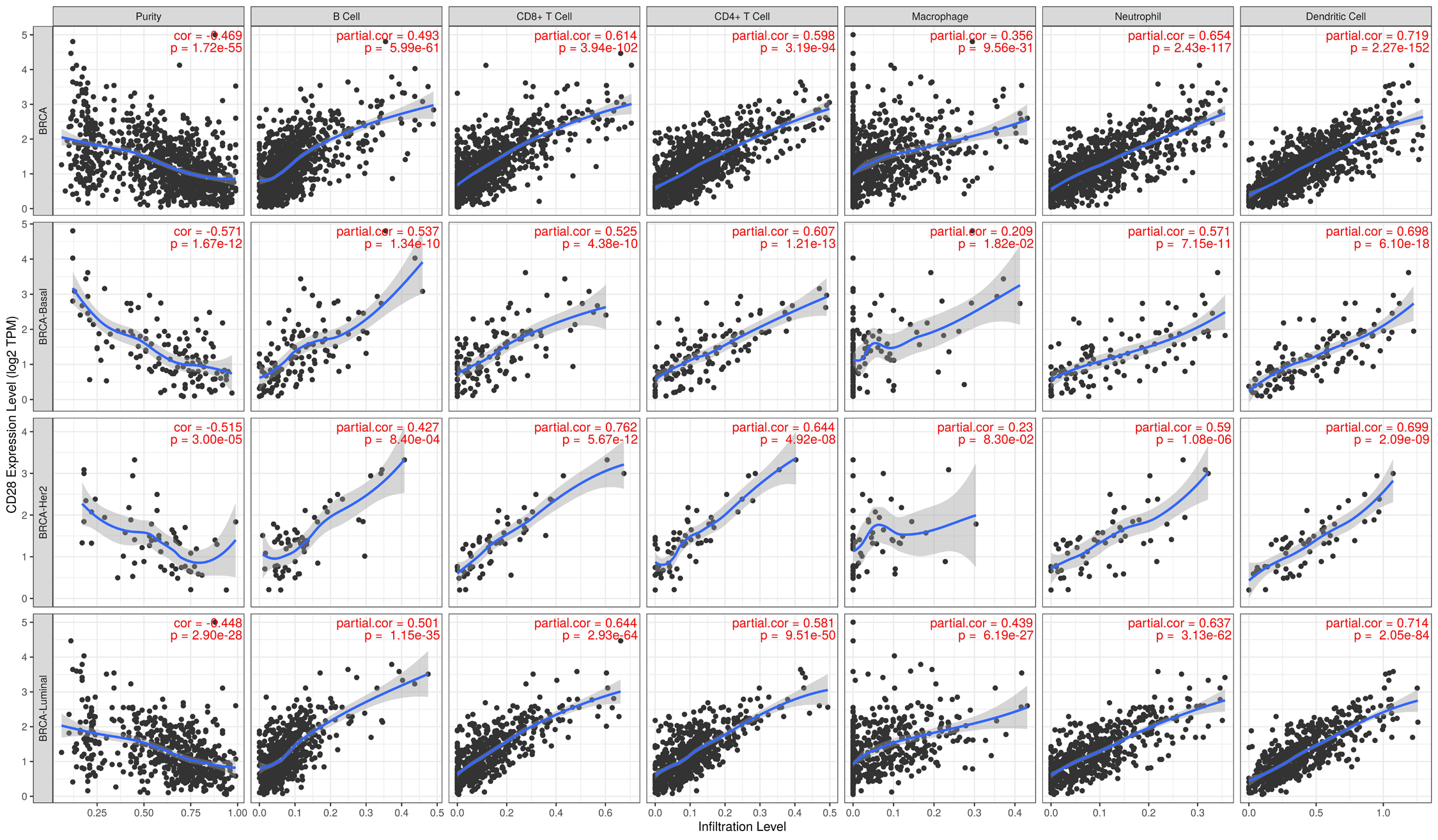

Supplement: S4 Fig — (TIF) [file pone.0344366.s004.tif]
